# Single molecule detection with graphene and other two-dimensional materials: nanopores and beyond

**DOI:** 10.1039/c5cs00512d

**Published:** 2015-11-27

**Authors:** Hadi Arjmandi-Tash, Liubov A. Belyaeva, Grégory F. Schneider

**Affiliations:** a Faculty of Science , Leiden Institute of Chemistry , Leiden University , Einsteinweg 55 , 2333CC Leiden , The Netherlands . Email: g.f.schneider@chem.leidenuniv.nl

## Abstract

Graphene and other two dimensional (2D) materials are currently integrated into nanoscaled devices that may – one day – sequence genomes. The challenge to solve is conceptually straightforward: cut a sheet out of a 2D material and use the edge of the sheet to scan an unfolded biomolecule from head to tail. As the scan proceeds – and because 2D materials are atomically thin – the information provided by the edge might be used to identify different segments – ideally single nucleotides – in the biomolecular strand. So far, the most efficient approach was to drill a nano-sized pore in the sheet and use this pore as a channel to guide and detect individual molecules by measuring the electrochemical ionic current. Nanoscaled gaps between two electrodes in 2D materials recently emerged as powerful alternatives to nanopores. This article reviews the current status and prospects of integrating 2D materials in nanopores, nanogaps and similar devices for single molecule biosensing applications. We discuss the pros and cons, the challenges, and the latest achievements in the field. To achieve high-throughput sequencing with 2D materials, interdisciplinary research is essential.

## Abstract 2

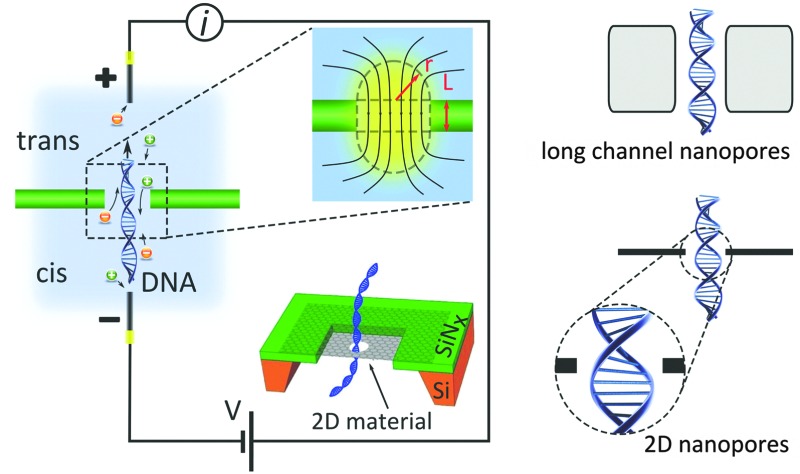
Graphene and other two dimensional (2D) materials are currently integrated into nanoscaled devices that may – one day – sequence genomes.

Key learning points

(1) [Mechanism] To learn the working principle of nanopores for biomolecule detection and sequencing.

(2) [General] To learn the advantages and the challenges of the implementation of 2D materials in nanopore schemes.

(3) [Mechanism] To learn about nanogap and the other proposals as the next generations of biomolecular sensors.

## 1 Introduction

Nanopore sensors have emerged as powerful devices for probing biomolecules. Head-to-tail sequencing is the ultimate goal of nanopore research. In comparison to existing sequencing technologies, nanopores potentially provide a single molecule platform for sequencing unlimited DNA lengths without chemical modifications or labeling. The working concept is straightforward: a DNA molecule is electrophoretically driven through a nanoscale pore in a membrane separating two aqueous environments. The signal (ionic current) corresponding to a nucleotide – momentarily present at the narrowest constriction of the nanopore – provides the information essential to identifying that specific nucleotide, primarily its molecular size or charge depending on the background ionic strength.

Ionic channels, naturally occurring in biological nanopores *e.g.* α-hemolysin and MspA, embedded in a lipid bilayer membrane were the first type of the nanopores experimentally probed, with now the proof that sequence information can be obtained.^1^ Solid-state nanopores, however, are more stable alternatives.^2^ The tunability of their physical and chemical properties, and their compatibility for mass-production are significant advantages.^2^

**Fig. 1:**
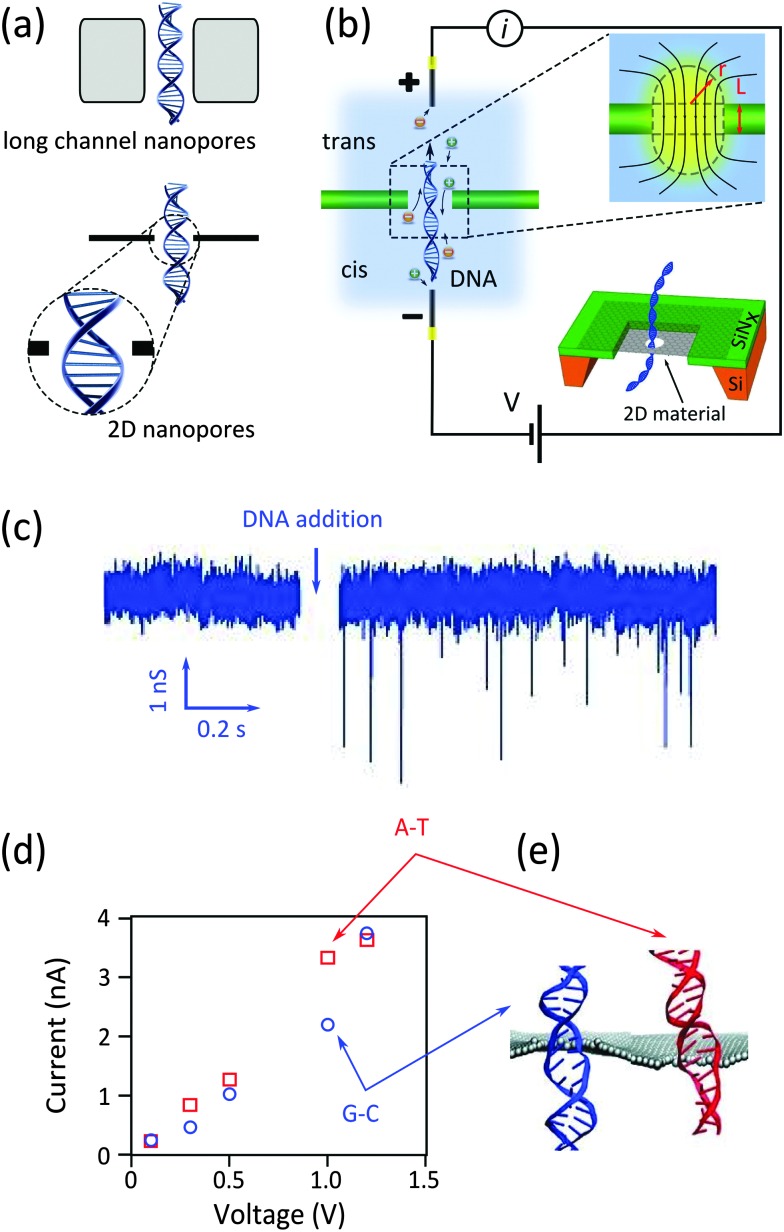
Nanopores for DNA detection. (a) Schematic representation of DNA translocation through long channel nanopores (membrane thickness ≫ spacing between nucleotides) and 2D nanopores (membrane thickness ≈ spacing between nucleotides). (b) Schematic representation of a typical nanopore device: an electrostatic field applied between two Ag/AgCl electrodes immersed in respectively the *cis* and *trans* reservoirs drives electrophoretically the ions and DNA molecules through the nanopore. The top inset figure shows the distribution of the field lines close to the nanopore. The dashed lines, composing a cylinder and two hemispheres, estimate the effective nanopore volume. The bottom inset figure illustrates the architecture of a 2D nanopore.^6^ (c) Measured current in a graphene nanopore system in the absence and presence of dsDNA in the buffer solution. Adapted with permission from ref. 3. Copyright 2010 American Chemical Society. (d and e) Simulated ionic current (d) and the conformations of poly(A–T)_20_ and poly(G–C)_20_ duplexes (e) passing through a 2D nanopore of *d* = 2.4 nm in graphene; adapted with permission from ref. 15. Copyright 2011 American Chemical Society.

The channel of a nanopore in a freestanding silicon-based membrane typically contains hundreds of bases simultaneously. Consequently such long channel nanopores are not capable of addressing and detecting single nucleotides composing a DNA strand. The integration of two-dimensional (2D) materials, especially graphene in nanopore systems, has drawn a lot of interest in recent years.^3–6^ As the thinnest possible materials with thicknesses comparable to the spacing between the nucleotides, monolayers of 2D materials promise very high spatial resolution, potentially eligible to DNA sequencing.^7^ Fig. 1a compares the resolution achievable by nanopores in silicon-based materials (long channel nanopores) and nanopores in 2D materials (2D nanopores).

Large, continuous and stable films of 2D materials can now be grown chemically to form free-standing membranes.^8–11^ Some of the 2D materials, particularly graphene, are electronically conductive with highly mobile and gate tunable charge carriers. Such properties open up new modalities for single molecule detection, *e.g.* by probing the doping effect of a translocating DNA molecule on the conductivity of graphene.^12^ Hexagonal boron nitride (h-BN) and molybdenum disulfide (MoS_2_) are the other 2D materials with demonstrated capabilities in nanopore applications.^13,14^

The far-too-rapid translocation of DNA molecules through nanopores, however, makes the detection of the individual nucleotides very challenging, both for 2D and long channel nanopores. Additionally, the low mechanical stability of atomically thin membranes is the Achilles' heel of 2D nanopores, adding considerable noise to the measured signal and reducing the achievable resolution. Due to such limitations, attempts to sequence DNA with 2D nanopores have not yet met the initial hopes. Instead, the platform has been extensively used to successfully detect the translocation of individual double-stranded^3–5^ and single-stranded^6^ DNA molecules.

Considering the rapidly growing interest in the application of 2D nanopores and nanogaps, this article serves as a comprehensive tutorial, reviewing the latest progress in the field. Background literature, fabrication methods and important achievements are presented in each section. We explain the remaining challenges and, importantly, offer appropriate solutions. This review bridges chemistry and physics and suggests new development routes in this still young and emerging research field.

## 2 Two-dimensional nanopores

2D materials and particularly graphene were recently integrated in nanopore sensors. Their mono-atomic thickness is at the center of the research interest. Although 2D materials conceptually promise single nucleotide resolution, they did not yet prove themselves as an alternative to commercially available Oxford Nanopore Technology sequencing platforms (www.nanoporetech.com).

### 2.1 Working principle of a nanopore

A nanopore is a nanoscale hole sculpted in a freestanding membrane (Fig. 1b). The membrane has a finite thickness *L* and separates two adjacent fluidic cells (*cis* and *trans*) containing a buffer solution. By applying a transmembrane potential (hundreds of mV), a constant ionic current is established as a result of the migration of ions between the cells. Upon the addition of single stranded or double stranded DNA molecules in the *cis* reservoir, the negatively charged DNA molecules are electrophoretically driven towards the *trans* reservoir. As single DNA molecules pass (*i.e.* translocate) through the nanopore, they partially block the flow of ions in the buffer, resulting in a series of sharp dips in the measured current (Fig. 1c). The statistical analysis of the current dips provides information about the structure – primarily the molecular size and charge – of the translocating molecule.

Identifying the building blocks of biomolecules (*i.e.* sequencing) is the driving force and the ultimate goal of nanopore analysis: theoretical calculations confirmed the possibility of distinguishing base pairs composing a double-stranded DNA (dsDNA) in graphene nanopore systems,^15^ as the ionic current largely depends on the conformation (stretching) of the translocating molecule. While the externally applied electrostatic field tends to stretch and deform base pairs occupying the nanopore, intermolecular hydrogen bonding in A–T and G–C base pairs (A: adenine, T: thymine, G: guanine, C: cytosine) resists against the deformation to some extent. Additionally, harsh electrostatic force can even break the hydrogen bonds and locally deform the molecules.^15^ The G–C base pair, however, is more robust against stretching than the A–T pair, as the former possesses an extra hydrogen bond. It was shown by computer simulation that an optimal driving force (∼1 V in this simulation) can sufficiently deform the A–T base pair so that the resulting ionic current can be distinguished from the G–C pair signal.

### 2.2 Fabrication technique

Two-dimensional materials, both in the form of mechanically exfoliated flakes or chemically grown large sheets, can be integrated in a nanopore device. Chemically grown macro-scale sheets are easier to manipulate; yet the crystalline quality of exfoliated flakes is superior. Achieving defect free membranes is vital as otherwise the migration of the electrolyte ions through the atomic scale defects (so-called “ionic current leakage”) can lead to the reduction of the signal to noise ratio.^4^

Exfoliated or chemically grown, the flakes have to be transferred onto a substrate with a previously drilled opening as shown in the bottom inset of Fig. 1b. The substrate provides a mechanical support for graphene while the opening in the supporting membrane allows biomolecules to translocate. Square or circular openings with sizes of hundreds of nanometers to few micrometers have been used^3–5^ (Table 2). The supporting membrane should be thin enough to effortlessly drill the opening, but with enough mechanical stability to support graphene. In practice, by partially etching a micro-scale window in the center of doped silicon wafers, free standing silicon nitride (SiN_*x*_) membranes with few tens of nanometers thicknesses are fabricated and used. Focused electron or ion beams are standard techniques to sculpt microscale openings in such membranes. In a next step, a 2D material is transferred onto the substrate covering the opening. The interface between the substrate and the 2D material should be well sealed against any ionic leakage to maximize sensitivity, for example using an elaborate transfer process.^16^ As the final step, a nanopore is drilled in the 2D material. Few techniques have been successfully used for drilling such nanopores.

#### 2.2.1 TEM sculpting

**Fig. 2:**
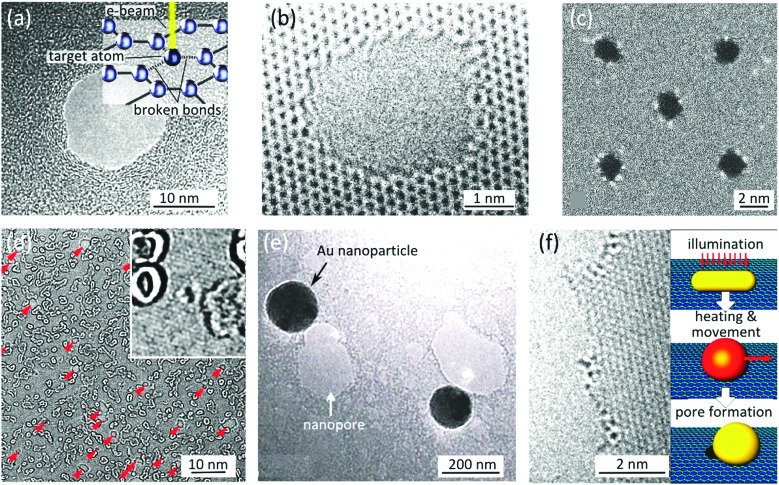
Methods for sculpting 2D nanopores. (a) TEM image of a nanopore sculpted with a high energy (300 kV) electron beam in a monolayer graphene at room temperature; adapted with permission from ref. 3. Copyright 2010 American Chemical Society. The inset schematically illustrates the process of breaking carbon–carbon bonds with such an energetic electron beam. (b) High resolution TEM image of a nanopore sculpted by high temperature STEM at 600 °C; adapted by permission from Macmillan Publishers Ltd: *Nat. Commun.*,^10^ copyright 2013. (c) TEM image showing an array of similar and small (*d* ≈ 2 nm) nanopores sculpted by STEM at 600 °C; adapted with permission from ref. 10. Copyright 2013 American Chemical Society. (d) TEM image of nanopores formed by argon ion bombardment followed by electron exposure: red arrows indicate the position of the randomly distributed nanopores. The inset shows a very small nanopore (*d* ≈ 5.8 Å) obtained with this technique. Images are adapted from ref. 21. (e) SEM image of nanopores photothermally formed in a graphene membrane: examples of gold nanoparticles and nanopores are visible in the image. Adapted with permission from ref. 22. Copyright 2014 American Chemical Society. (f) High resolution TEM image of an edge of a nanopore formed in graphene using the photothermal method: The inset schematically describes the processes leading to the pore formation. Adapted with permission from ref. 22. Copyright 2014 American Chemical Society.

Transmission electron microscopy (TEM) is the most used technique for sculpting nanopores and arrays of nanopores in graphene and other 2D materials.^3–5,9,12^ The bonds between the atoms in the 2D crystal are mechanically broken by collisions of highly focused and accelerated electrons (inset of Fig. 2a). Furthermore, and if required, drilling both the graphene and the supporting SiN_*x*_ membrane can be done simultaneously.^12^ The just-sculpted structures can be readily imaged in the scanning mode of the TEM. The formation of defects and amorphization close to the nanopore in monolayer graphene membrane (less likely to happen in few layer membranes^17^) is a common problem in the normal application of the technique. Additionally it is shown that organic process residuals and contaminations can migrate to the beam spot and accumulate, locally raising the effective thickness of the edge far beyond the atomic thickness of the membrane.^18^ Fig. 2a shows a TEM image of a 2D nanopore sculpted using this method.^3^

#### 2.2.2 High temperature STEM sculpting

By optimizing sculpting parameters, it was demonstrated that the working temperature has an important effect on the crystallinity of sculpted nanopores:^18^ *in situ* annealing (*T* ≥ 600 °C) during electron beam sculpting in TEM can effectively prevent contamination and amorphization of graphene. Remarkably, high-resolution TEM images confirmed that the crystallinity of graphene is preserved up to the edges (Fig. 2b). In a more advanced method using a scanning transmission electron microscope (STEM),^10^ very fine nanostructures (*e.g.* nanopores of *d* ≈ 2 nm) were sculpted with predefined positions and sizes (Fig. 2c). Electrophysiological measurements on those nanopores showed that the effective length of the nanochannel is comparable to the thickness expected for monolayer graphene.^19^ Preserving the crystallinity of graphene up to the edges is particularly important in applications where the nanopore rim is chemically functionalized. However, the high costs involved in TEM and STEM techniques and their incompatibility with scalable device fabrication intrinsically limit their application into commercially viable devices.

#### 2.2.3 Atom by atom growth of nanopores

In conventional TEM, the electron beam has to possess enough energy (*E* > 80 keV^20^) to break three carbon–carbon bonds in the graphene lattice to nucleate a defect (inset of Fig. 2a). Once nucleated, the defect grows very fast since lower energies are required to knock out atoms with already broken bonds. The atom-by-atom growth technique disentangles the nucleation and the growth phases:^21^ first, a low energy (3 keV) Ar^+^ ion beam nucleates arrays of vacancy defects which are subsequently grown with unfocused subthreshold energy electron beams. Nanopores with diameter as small as 6 Å were obtained with this approach (inset of Fig. 2d). While the technique is efficient for making sub-nanometer nanopores, further developments are still required to provide control over the position, shape and number of the achieved nanopores.

#### 2.2.4 Photothermal nanopore formation

Photothermal nanopore formation is another scalable technique yielding arrays of nanopores at room temperature.^22^ Monodisperse gold nanorods are first drop-casted on a graphene membrane. Then a femto-second laser with a wavelength close to the geometry-related maximum absorbance of the nanorods illuminates the surface of the sample. As a result of the plasmonic character of the metallic nanorods, light energy converts into heat. Under the dramatic increase in temperature (up to 680 °C^22^), the nanorods deform into nano-hemispheres which are mobile under the light source. The temperature in the vicinity of the nano-hemispheres largely exceeds the oxidation temperature of graphene, finally yielding nanopores. Fig. 2e and f show the process flow and the achieved nanopores. The size and shape of the nanopores are customizable by fine-tuning the illumination parameters and initial size of the nanorods. The process partially preserves edge crystallinity, as one can judge from the high resolution TEM images (Fig. 2f). The gold nanoparticle at the border of the nanopore can also be useful as plasmonic sensors (will be discussed in Section 2.5). Mass production of nanopores is another advantage of the technique. Nevertheless, the geometries of the nanopores are not precisely controllable and automated. Furthermore, the drop-casting method leads to random localization of gold nanorods and therefore of the formed nanopores. Separately, the presence of gold particles in the vicinity of the nanopores is likely to increase the effective channel length, working against the initial idea of using monoatomic membranes.

#### 2.2.5 Summary of the fabrication methods

Table 1 summarizes the advantages and drawbacks of the techniques used for sculpting nanopores in graphene and other 2D materials. The atom by atom growth of nanopores provides the smallest nanopores; the process, however, fails to control the number, position and geometry of nanopores. The photothermal formation of nanopores suffers from similar drawbacks. The TEM technique, however, permits control over the position of the nanopores; although very often large defects (holes) form beside the nanopores. The position, size and geometry of nanopores are precisely controlled in the high temperature STEM mode. Moreover, the crystallinity of the edges also is preserved. However, the time-consuming sequential STEM sculpting of arrays of nanopores (∼5–10 minutes per nanopore) limits the throughput of the technique for industrial applications. We note that the techniques listed in Table 1 have been mostly applied for sculpting graphene. For other 2D materials, TEM has been primarily used (h-BN^13^ and MoS_2_^14^).

**Table 1:** Pros and cons of the different methods used for patterning nanopores

Technique	Smallest nanopore	Crystallinity	Parallel^*a*^	Drilling SiN_*x*_ ^*b*^	Reproducibility	Ref.
TEM	*d* ≈ 2 nm	Destroyed	No	Yes	Poor	3–5, 9 and 12
High-*T* STEM	*d* ≈ 2 nm	Preserved	No	Yes	Yes	10 and 19
Atom by atom growth	*d* ≈ 6 Å	Not clear	Yes	No	No	21
Photothermal	2 × 60 nm^2^	Partially preserved	Yes	No	No	22

^*a*^The possibility for sculpting several nanopores at the same time.

^*b*^The possibility for drilling SiN_*x*_ membrane simultaneously with the 2D material.

**Table 2:** Translocation speed of DNA in 2D nanopore systems

Molecule	Membrane		Geometry		Translocation speed^*a*^	Ref.
	Material	# Layers	Opening	Nanopore (nm)		
dsDNA	Exfoliated graphene	1–8	*d* = 5 μm	5–25	56 ns per bp, 22 nm, 200 mV	3
dsDNA	CVD graphene with/without 5 nm of TiO_2_	3–15	*d* = 1.5 μm	5–10	30 ns per bp, 8 nm, 100 mV 14 ns per bp, 6 nm, 100 mV^*b*^	4
dsDNA	CVD graphene	1–2	200 × 200 nm^2^	5–23	Not reported	5
ssDNA & dsDNA	CVD graphene	1	200 × 200 nm^2^	3–7	10 ns per bp, 3.3 nm, 160 mV	6
dsDNA	CVD h-BN	2–3	300 × 300 nm^2^	5–14	20 ns per bp, ∼6 nm, 100 mV 16 ns per bp, ∼6 nm, 160 mV	13
dsDNA	CVD graphene with 5 nm Al_2_O_3_	1	n/a	10	n/a	12
ssDNA	Exfoliated graphene	1	*d* = 1.5 μm	3–20	∼19 ns per nt, 15 nm^*c*^, 200 mV ∼26 ns per nt, 10 nm^*c*^, 200 mV ∼36 ns per nt, 5 nm^*c*^, 200 mV	19
dsDNA	Exfoliated MoS_2_	1–4	200 × 200 nm^2^ up to 500 × 500 nm^2^	2–20	∼20 ns per bp, 20 nm, 200 mV	14

^*a*^The translocation speed is calculated based on the reported translocation time for an unfolded DNA. The reported data in this column are respectively: the translocation speed, the diameter of nanopore and the applied transmembrane voltage.

^*b*^The membrane is covered with 5 nm of TiO_2_.

^*c*^Covering the surface of graphene and the perimeter of the nanopore with hydrophilic layer makes the effective nanopore diameter smaller.

### 2.3 Nanopore implementation

So far 2D nanopores have been extensively used for probing the translocation of single and double stranded DNA. No successful implementation of 2D nanopores for DNA sequencing has been reported yet. Several parameters affect the performance of a nanopore, which will be discussed in this section and some are summarized in Table 2.

#### 2.3.1 DNA conformation

**Fig. 3:**
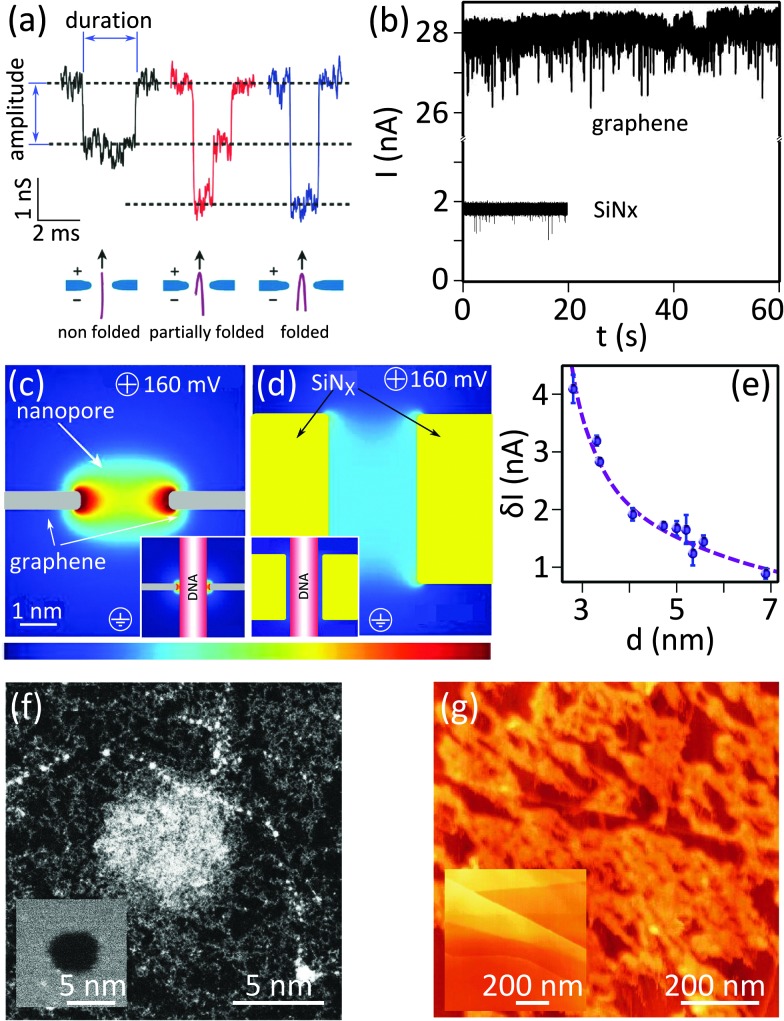
Implementation of 2D nanopores for detecting biomolecules. (a) Examples of typical conductance signals for DNA translocating through nanopores: the color coding depicts the different folding configurations. Adapted with permission from ref. 3. Copyright 2010 American Chemical Society. (b) Ionic conduction through nanopores of similar diameters in graphene (*d* ≈ 8 nm) and SiN_*x*_ (*d* ≈ 6 nm, *L* = 40 nm) membranes. The experiments were both performed in 1 M KCl, 10 mM Tris (pH = 8.1). Numerous translocation events are visible as sharp current dips in both time traces. Adapted with permission from ref. 4. Copyright 2010 American Chemical Society. (c and d) Graphical representation of simulated ionic current density through a (c) 2D nanopore (*d* ≈ 2.5 nm, *L* = 0.6 nm) and (d) a long channel nanopore (*d* ≈ 2.5 nm, *L* = 5 nm); insets show the same simulation in the presence of a translocating dsDNA strand (modeled as insulating cylinder with *d* = 2.1 nm). The color coding represents the current density and ranges from *j* = 0 to 2 GA m^–2^ and *j* = 0 to 4 GA m^–2^ for the main and inset figures respectively. The figure is adapted from ref. 6. (e) Influence of the diameter of nanopores in single-layer graphene on the average current blockade (*δI*); the dashed line shows results originating from simulation. The figure is adapted from ref. 6. (f) STEM image of a 2D nanopore in graphene (*d* = 5 nm) before (inset) and after (main panel) the incubation with single stranded circular M13 DNA; the white blob at the center of the nanopore is presumably a DNA molecule clogging the nanopore. Adapted by permission from Macmillan Publishers Ltd: *Nat. Commun.*,^19^ copyright 2013. (g) AFM images of the surface of HOPG before (inset) and after (main panel) incubation with M13 ssDNA; adapted by permission from Macmillan Publishers Ltd: *Nat. Commun.*,^19^ copyright 2013.

An example of the electrical signal measured from a nanopore is shown in Fig. 1c. The DNA translocation events can be recognized as sharp dips in the current. Fig. 3a focuses on a few of such events. Each translocation event is characterized by (i) the event duration (the width of the dip), and (ii) the amplitude of the current blockade (the depth of the dip). The speed of DNA translocation through the nanopore defines the duration of the event: the faster the translocation, the sharper the dip. The magnitude of the current blockade indicates to what extend the molecule obstructs the flow of the ionic current through the nanopore. Molecules can translocate through the nanopore in different macroscopic configurations. The translocation of a fully extended molecule leads to a single-dip event (black signal in Fig. 3a). Many dips with amplitudes doubling such events (blue signal) or the combination of those (red signal) are normally present in a typical recorded time-trace. They are attributed to the translocation of fully or partially folded DNA molecules respectively. The bottom panel in Fig. 3a illustrates the typical conformations of the translocating DNA molecules. We note that nanopores with diameters below ∼4 nm are too small to admit folded dsDNA molecules; only unfolded dsDNA can pass through such nanopores.^6^

#### 2.3.2 Geometry of nanopores

The transmigration of ions through a nanopore is accompanied by the ionic resistance, which is affected by the effective geometry of the nanopore. In a simple model, a nanopore with a diameter *d* = 2*r* in a membrane of thickness *L* can be regarded as a cylinder:^23^ The conductance increases with increasing nanopore diameter and decreases with increasing channel length, which can be obtained by selecting thicker membranes. This model is mainly valid when *L* > *d* (thick nanopores). For nanopores in thin materials *e.g.* 2D nanopores, the convergence of the electric field lines at the “mouth” of the nanopores (inset Fig. 1b) increases the effective nanopore thickness beyond the membrane thickness. Correction for the effective volume of thin nanopores are normally represented by two hemispheres on both sides of the nanopore;^23^ the corresponding impedance is referred to as the “access resistance”. The dashed lines in the top inset in Fig. 1b show the estimated effective geometry of a nanopore.

A general model for the conductance of a nanopore (*G*) is proposed as:^23,24^
1
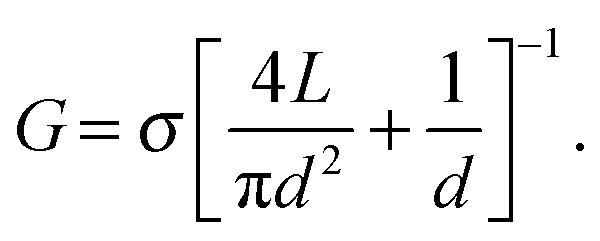
 Here *σ* refers to the ionic conductivity of the buffer solution. The first and the second terms show the nanopore channel resistance and the access resistance respectively. For ultra-thin membranes (*L* ≪ *d*), the first term approaches zero and the access resistance dominates; hence the conductance increases linearly with the nanopore diameter: *G* ∝ *d*. The coefficient of proportionality depends on the concentration of the ions and their mobilities. In contrast, when the thickness of the membrane is comparable to or larger than the nanopore size, the ionic conductance scales quadratically with the nanopore size: *G* ∝ *d* ^2^.

Fig. 3b compares the ionic current measured from a 2D nanopore in graphene (*d* = 8 nm) and from a nanopore with a similar diameter (*d* = 6 nm) in a SiN_*x*_ membrane of *L* = 40 nm.^4^ The baseline current in the 2D nanopore is several times larger than the one in the SiN_*x*_ membrane. This observation is consistent with eqn (1) since the reduction of the membrane thickness yields a higher conductance. Indeed, ions feel much less resistivity passing through nanopores in 2D materials, compared to long channel nanopores.

Although achieving higher resolution is an important motivation for using 2D nanopores, some reports argue about the efficiency of using monolayer membranes.^25^ Molecular dynamic simulations showed that once a negatively charged ssDNA enters the nanopore, the change of the ion distribution results in an electric double layer near the surface of the DNA strand, reducing the sensitivity of the ionic current to the size of the bases. The effect is stronger in thin (monolayer) graphene membrane; hence the relatively thick membranes (few layer membranes) are claimed to be more efficient for sequencing ssDNA.

#### 2.3.3 Sensitivity of the nanopores

The mechanical instability of the membrane in which the nanopore is sculpted greatly affects the spatial resolution. As freestanding 2D materials are mechanically less stable than thick membranes, their mechanical instability is more of a concern. In a 2D nanopore, the amplitude of the membrane vibrations may reach a level comparable to the thickness of the membrane.^15^ Induction of low frequency noise on the measured signal is another drawback of the instabilities.^6^

Several approaches can reduce the mechanical vibration of 2D nanopores in buffer solutions. The process for the fabrication of free standing membranes may induce some mechanical stress in the final membrane which limits the mechanical fluctuations. Additionally, the concentration of salt in the buffer solution also affect the mechanical vibrations: the more concentrated the salts, the more ions colliding the membrane and the less mechanical stability.^19^ Increasing the working temperature also has similar consequences.^26^

Additionally, the spacing between the border of the nanopore and the DNA (*i.e.* the relative size of the nanopore *versus* the size of the DNA molecule) is another parameter affecting the sensitivity of nanopores. Simulations demonstrated that in long channel nanopores, the ionic current density is uniformly distributed throughout the channel width as the edges of 2D nanopores strongly localize the ionic current^6^ (Fig. 3c and d). Therefore, when the diameter of the nanopore approaches the diameter of the translocating DNA (insets of Fig. 3c and d), the localized current is more sensitive to the spatial spreading of the DNA building blocks. Indeed, sub-nanometer resolution and ultra-high sensitivity to extremely small changes in the local diameter of the translocating molecule can be achieved with a tightly dimensioned nanopore. The results were confirmed experimentally (Fig. 3e).^6^

#### 2.3.4 Sustainability of 2D nanopores

Translocation experiment with clean and crystalline membrane is challenging: DNA sticks strongly (hydrophobic interaction) on the membrane and may finally clog the nanopore (Fig. 3f). AFM mapping of a graphitic surface exposed to the solution containing single stranded DNA confirmed the strong adsorption of DNA on the surface (Fig. 3g);^19^ the results are in agreement with theoretical predictions.^27^ The irreversible adsorption is expected to be driven by aromatic purine and pyrimidine bases in DNA molecules. Several approaches have been employed to minimize the interaction.

A rapid UV/ozone treatment can turn graphene into graphene oxide with hydrophilic properties. The oxidation by itself may not be enough as 30 percent of the devices measured after the treatment^4^ did not show enough wettability to translocate DNA. Instead, depositing a thin layer of hydrophilic TiO_2_ films on both sides of the graphene membrane further improved the wettability of the devices.

The composition of the buffer solution is another parameter affecting the interaction between the molecule and the graphene surface. Salty solutions (3 M KCl) and high pH of the buffer solution (up to 10) minimize the interaction of DNA with the surface, leading to faster DNA translocation.^6^ Note that a further increase in the pH (up to ∼12) may denature dsDNA into ssDNA. In a separate study, 3M of KCl did not prevent DNA from interacting with graphitic surfaces:^19^ AFM mappings showed that ssDNA still sticks rather strongly to the surface of HOPG (Fig. 3g). Instead, a more general approach based on non-covalently functionalizing graphene with hydrophilic ethylene glycol groups reduced DNA–graphene interactions.^19^ A reduced hydrophobic interaction between the aromatic graphene and nucleotides, at the cost of just a 1–2 nm increase in the channel length, was demonstrated upon coating graphene with such an ultrathin layer.

#### 2.3.5 Pin-holes in the membrane

Pin-holes, nanometer scale defects in 2D crystals, create parallel and unfavorable paths for the migration of the ions (rather than through the nanopore), and thus reduce the sensitivity of the measurement. Such pin-holes may form during the growth or transfer of the 2D materials or because of the post treatment used to modify the hydrophobicity of the surface. As a result, the blockade current in the nanopore could be one order of magnitude less in defected CVD graphene compared to nanopores with the same diameter in silicon nitride membranes.^4^ Experimental results showed that even coating graphene with 5 nm thick TiO_2_ film could not block such parallel paths effectively.^4^ Minimizing the suspended graphene area could be an effective method to minimize the contribution of the pin-holes to the total ionic current.

### 2.4 Major challenges to reach DNA sequencing with 2D nanopores

It is now well established that the detection of the translocation of entire DNA molecules in solid-state nanopores is possible. Attempts, however, to identify individual nucleotides within a DNA strand have not yet succeeded. The rise of 2D materials in nanopore technology raised hopes for achieving single base resolution. Despite the considerable efforts over the last five years, high throughput sequencing has not been realized yet with 2D materials. This section reviews the drawbacks and challenges.

#### 2.4.1 Fabrication of atomically-thin nanopores

**Fig. 4:**
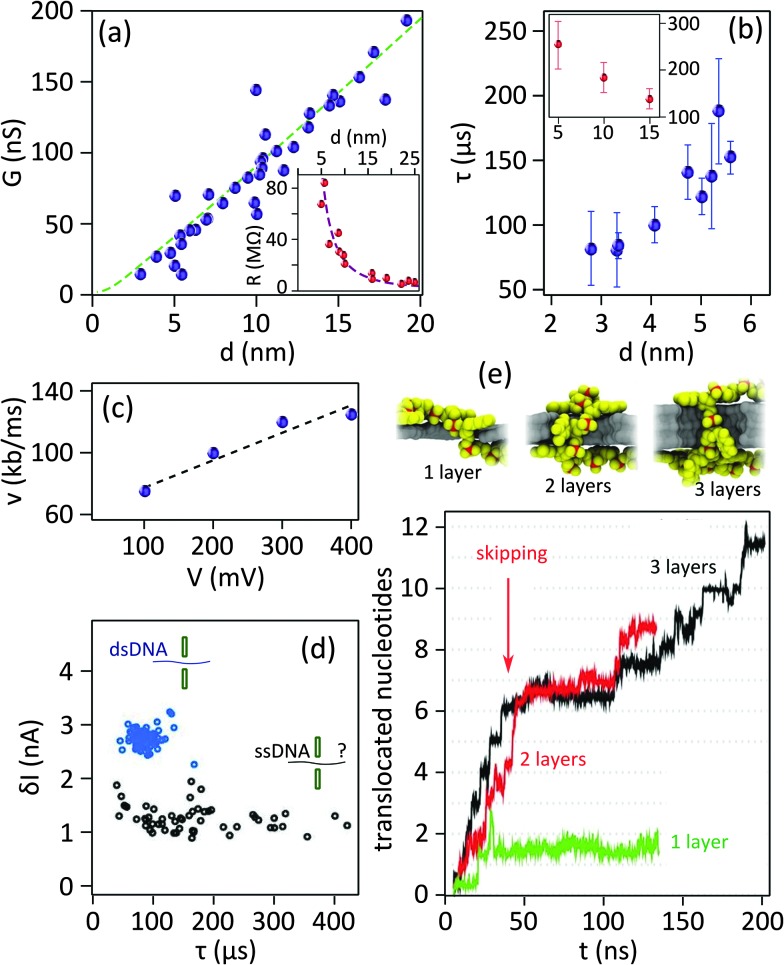
Important challenges of 2D nanopores for sequencing biomolecules. (a) Conductance of 2D nanopores in graphene as a function of the diameter of the nanopore: the data in the main panel is measured from nanopores sculpted using high temperature STEM. Dashed green line represents the best fitting with eqn (1) revealing *L* = 1.2 ± 0.1 nm. Adapted by permission from Macmillan Publishers Ltd: *Nat. Commun.*,^19^ copyright 2013. The inset shows the resistance of 2D nanopores in graphene membranes (number of layers ranging between 1 to 8) sculpted by room temperature TEM as a function of the diameter. The best fitting with eqn (1) reveals a membrane thickness of 9 nm. Adapted with permission from ref. 3. Copyright 2010 American Chemical Society. (b) Translocation time as a function of the diameter of 2D nanopores in graphene membranes: different experiments (blue and red) compared in the main^6^ and inset^19^ panels show opposite trends. The vertical and horizontal axes in the inset have the same unit as the main panel. The inset panel is adapted by permission from Macmillan Publishers Ltd: *Nat. Commun.*,^19^ copyright 2013. (c) Translocation speed of dsDNA through a graphene nanopore with a diameter of 8 nm plotted as a function of the transmembrane potential: the membrane is composed of a graphene coated with 5 nm of TiO_2_. Dashed line shows the linear fit of the data. Adapted with permission from ref. 4. Copyright 2010 American Chemical Society. (d) Current blockade amplitudes plotted as a function of the translocation time for double stranded (blue circles, measured at pH = 10) and single stranded (black circles, measured at pH = 12.5) DNA molecules. The data were measured in a graphene nanopore of *d* = 3.3 nm. The dsDNA molecules translocate through the nanopore in a fully unfolded configuration. The data are adapted from ref. 6. (e) Simulation of the translocation of ssDNA through 2D nanopores in one-, two-, and three-layer graphene membranes: (top) Snapshots showing translocating ssDNA (poly(dT)_20_) through membranes with different number of layers, (bottom) plot represents the number of translocating nucleotides as a function of time (*t*). For single layer graphene (green) the whole ssDNA translocates in a single step, while for two- and three-layer membranes multiple steps are observed (red and black). The data are simulated for a 500 mV bias voltage. Adapted with permission from ref. 27. Copyright 2012 American Chemical Society.

Whereas eqn (1) predicts a linear dependency of the conductivity *versus* nanopore diameter for 2D nanopores, early experimental results^3^ depicted a quadratic relation which is the expected behavior for a cylindrical and long channel nanopore (inset of Fig. 4a). Indeed the best fitting of the data with eqn (1) revealed a channel length of *L* = 9 nm. Deposition of carbon atoms close to the border while drilling the nanopore can be an explanation for the observation.^18^ The finally achieved thick edges ruin the advantage of using 2D materials in nanopores by highly limiting the resolution of the device. *In situ* annealing of the sample during the sculpting has solved the problem,^18^ achieving an effective channel length of *L* = 1.2 ± 0.1 nm^19^ (Fig. 4a).

#### 2.4.2 High translocation speed

To be able to differentiate between nucleotides composing single-stranded DNA (ssDNA), the translocation must be performed in single-nucleotide steps:^27^ a nucleotide unbinds (hydrophobic interaction) from one side of the membrane and binds to the other side after a small sliding of the strand along the surface. Simulations predict a time span of 16 ns for each nucleotide (nt) to pass through a graphene nanopore (*d* = 16 nm, bias voltage = 500 mV).^27^ Experimentally, translocation times ranging between 10 ns^6^ to ∼60 ns^3^ per base (pair) have been reported in different conditions and for single and double stranded DNA (Table 2).

The bandwidth of the measurement electronics sets an upper limit to the achievable temporal resolution. The combination of the membrane with the ions at its both sides can be regarded as a parallel plate capacitor which adds high frequency noise to the measured signal. To attenuate this noise, it is inevitable to use a low-pass filter of ∼10 kHz as a part of the electronic detection scheme.^28^ Using such a filter sets a maximum temporal resolution of ∼50 μs. The achieved resolution is therefore much inferior to the required resolution to resolve single nucleotides.

Slowing down the translocation speed of the DNA is an important objective followed by many researchers, particularly when using biological nanopores. In fact, a DNA translocating through a nanopore experiences three different forces, namely the electrophoretic driving force, the nanopore-molecule interaction force, and the drag (viscosity) force from the buffer solution. While the electrophoretic driving force accelerates the translocation of DNA, the two others impede against the translocation. Several parameters modulate those forces and therefore the translocation speed of DNA; they will be discussed in the following sections.

##### 2.4.2.1 Effects of the nanopore geometry

A correlation between the translocation speed and the nanopore diameter has been reported experimentally:^6^ the smaller the nanopore, the faster the translocation. The observation was explained in terms of the decreased drag on the translocating molecule in smaller nanopores. However it seems this effect is not universal since a contradictory trend was also reported elsewhere for 2D nanopores^19^ (Fig. 4b, main and inset panels). This observation can be attributed to the nanopore–DNA interaction.

The shape of the nanopore may also affect the translocation speed: in circular nanopores, increasing the bias voltage generally accelerates the translocation. However simulations show that broken rotational symmetry in elliptical nanopores affects the DNA atomic conformation inside the nanopore: DNA molecules may reshape in conformations which are unfavorable for translocation, slowing down the molecules.^27^

##### 2.4.2.2 Electrostatic force on translocating DNA

The electrostatic force produced by the transmembrane potential is the most important parameter governing the translocation speed of the DNA. In-fact, a linear dependency of the translocation speed *versus* transmembrane voltage was reported experimentally^4^ (Fig. 4c). Therefore, minimizing the transmembrane potential is the most direct approach to slow down the DNA. We note that the transmembrane potential largely controls the ionic current: diminishing the signal-to-noise ratio is an important drawback of this approach. Transmembrane potentials ranging between 100–200 mV have been typically used in the experiments so far (Table 2).

Manipulating the net charge on the DNA is the more efficient method to modulate the translocation speed. Ions in the buffer, such as magnesium chloride, can coordinate on the DNA and strongly screen the net charges.^29^ Additionally, protons in the solutions with low pH values can partially neutralize the negative charge on the DNA molecules and reduce the translocation speed.^30^

##### 2.4.2.3 DNA–membrane interaction

Considering that the nanopore is of very small size in comparison to the rest of the membrane area, DNA molecules adsorb on the surface of the membrane before translocating through the nanopores. The DNA molecules can be very mobile and diffuse at the surface to ultimately reach the nanopore.^27^ In this process, the interaction between the hydrophobic DNA and the membrane would largely impact the translocation speed.

Single stranded DNA shows stronger hydrophobic interaction with hydrophobic graphene as the coupling of the two strands in a dsDNA minimizes the overall hydrophobicity of the molecule.^27^ Fig. 4d compares the translocation events of double stranded and single stranded DNA through a 2D nanopore in graphene of *d* = 3.3 nm.^6^ As a result of the ultra-small area of the nanopore, only unfolded dsDNA can translocate. Double stranded DNA shows uniform translocation characteristic since the events possess very similar durations. In contrast, the translocation time for ssDNA ranges from ∼50 μs up to more than 400 μs. Strong hydrophobic interaction between graphene and ssDNA can explain the divergent translocation times.

A more detailed study about the DNA–membrane hydrophobic interaction was performed by probing the translocation of single and double stranded DNA through nanopores in graphene–Al_2_O_3_ stacked membranes.^31^ Different configurations of stacking layers, including graphene/Al_2_O_3_/graphene, Al_2_O_3_/graphene and standalone Al_2_O_3_, have been examined. The results are summarized in Table 3. Translocation of the ssDNA is slower through membranes with graphene layers compared to standalone Al_2_O_3_, pointing out the strong hydrophobic interaction between graphene and ssDNA. The fact that a double stranded DNA molecule exhibits much faster translocation compared to single stranded DNA in similar membranes (graphene/Al_2_O_3_/graphene) is another confirmation that in ssDNA purines and pyrimidines are more accessible for pi-stacking interaction with graphene.

**Table 3:** Translocation rate of single and double strand DNA through nanopores in graphene and Al_2_O_3_ multi-stacked membranes

	Membrane configuration	Translocation rate^*a*^
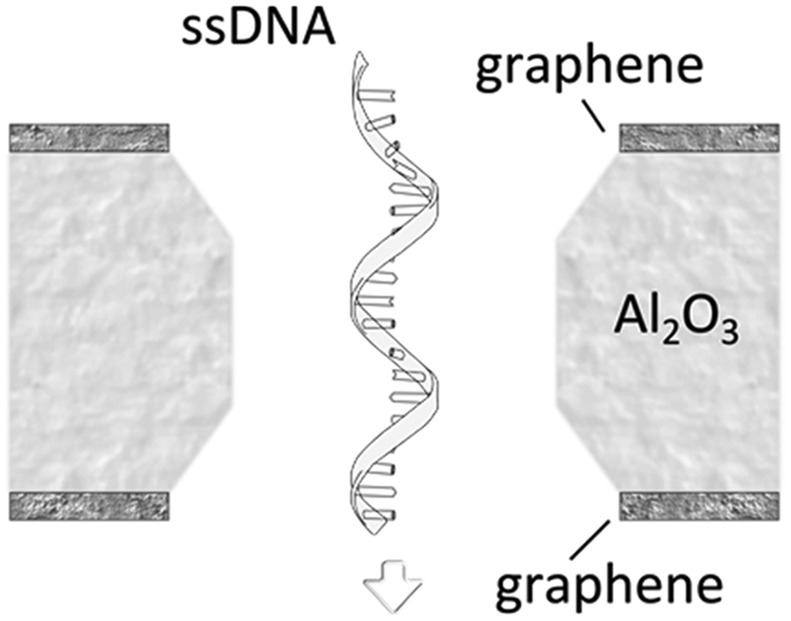	Graphene–Al_2_O_3_–graphene (hydrophobic–hydrophilic–hydrophobic)	5.5 μs per nt (for ssDNA)
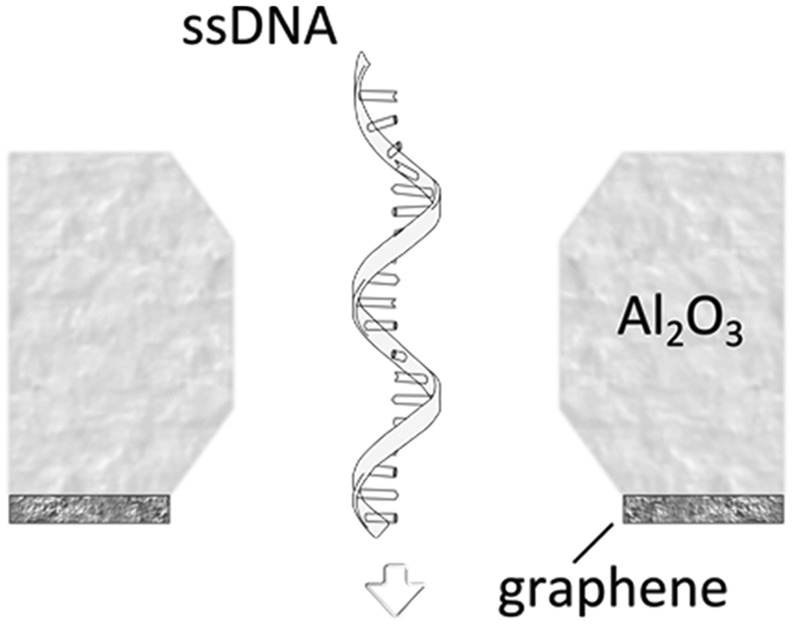	Al_2_O_3_–graphene (hydrophilic–hydrophobic)	4.7 μs per nt (for ssDNA)
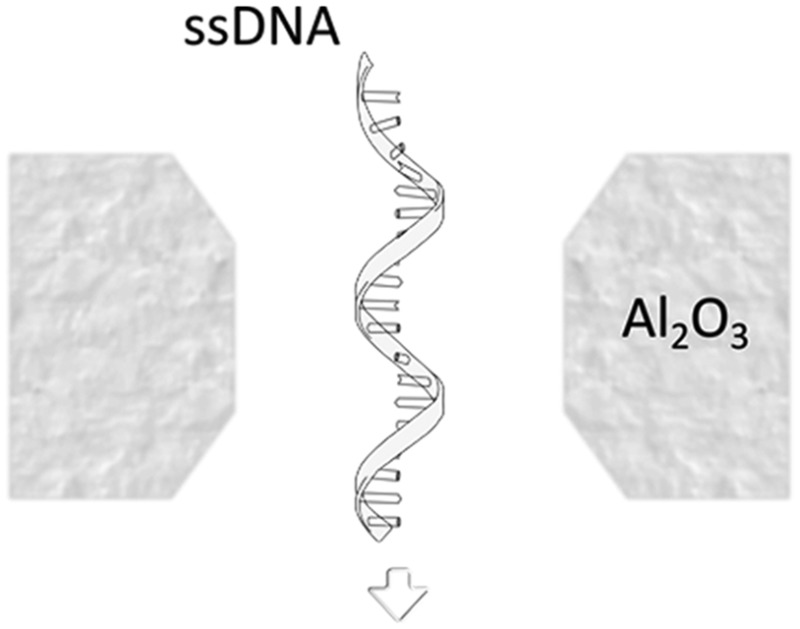	Al_2_O_3_ (hydrophilic)	1.8 μs per nt (for ssDNA)
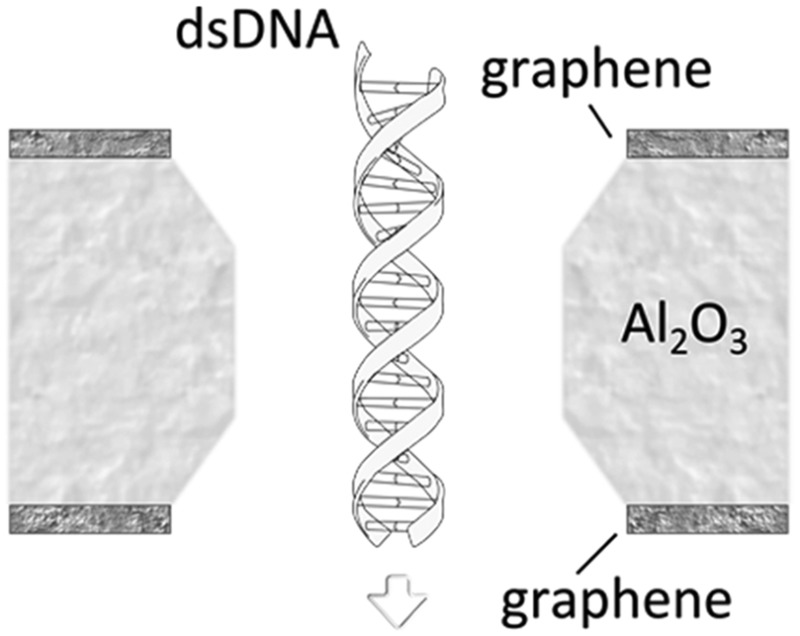	Graphene–Al_2_O_3_–graphene (hydrophobic–hydrophilic–hydrophobic)	0.4 μs per bp (for dsDNA)

^*a*^The translocation is measured at 300 mV bias and for nanopres with similar diameters.

In addition to the naturally occurring hydrophobic interaction between the DNA and 2D materials, electrostatic interaction also can be induced. Chemically functionalizing the nanopore with positively and negatively charged groups is a way to achieve nanopores with different charge states: as DNA is also charged (naturally), the DNA–membrane electrostatic interaction can affect the translocation speed. Simulations illustrated that the translocation through a nanopore (*r* = 1.2 nm) in a positively charged membrane (+3.6*e*) can happen almost twice faster than a similar nanopore in a negatively charged membrane (–3.6*e*).^15^ Indeed the repulsive interaction between the negatively charged DNA molecule and nanopore rim reduces the effective nanopore diameter.

Electrically biasing the conducting 2D membrane is another approach to induce (modulate) an electrostatic interaction. Stacked membranes in which an electrically connected graphene membrane is sandwiched between two dielectric materials is a model systems for this purpose.^20^ Even though the reduced translocation speed is a favorable consequence of strong DNA–membrane interaction, such interactions should still be avoided: the interaction can reduce the stability of the device by clogging the nanopore.^19^ Increasing the noise level is another drawback.^4^

##### 2.4.2.4 The length of the fluidic channel

The effects of the fluidic channel length – set by the thickness of the membrane – on the translocation speed have been studied both theoretically and experimentally. The total electrical force (*F*) felt by the DNA molecule translocating through the nanopore depends on the externally applied electrical field (*E*) and the electrical charge of the molecule inside the nanopore (*q*): *F* ∝ *Eq*. While a larger electric field (
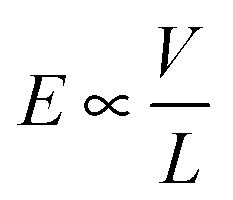
, *V*: transmembrane potential, *L*: membrane thickness) is established across thinner membranes, the total negative electrical charge on the portion of the DNA residing inside the thinner nanopore is smaller. Consequently, in theory, no strong effect of the channel length on the total electrical force and hence on the translocation speed is predicted.

Note that even if the translocation speed remains the same in long and short channels, the time that a DNA nucleotide occupies the channel in longer channels is larger; hence enhanced temporal resolution can be achieved. Indeed, simulations show that single-nucleotide steps could be identified in bi- and three-layer graphene nanopores^27^ (Fig. 4e). The co-presence of multiple bases in the nanopore is the drawback limiting the spatial resolution.

Experimentally, nanopores drilled in few layer graphene yielded slower translocations (more than twice as slow) compared to similar nanopores drilled in 20 nm-thick SiN_*x*_ membranes.^3^ Similar comparison with hexagonal boron nitride (h-BN) membranes^13^ reveals an opposite trend *i.e.* faster translocation through h-BN nanopores in comparison than in SiN_*x*_ ones. Such contradictionary observations may also point out the influence of chemical composition of the membrane material on the strength of the DNA–membrane interactions.

#### 2.4.3 Conformational instabilities of biomolecules

Nucleotides can take diverse molecular conformations while passing through nanopores. Examples of such conformations are depicted in the snapshots in the top panels of Fig. 4e. As the ionic conduction depends on the local three-dimensional extension of the translocating molecule, the variation of the nucleotides' conformation is a source of inaccuracy for sequencing applications. Simulations demonstrated that improving the DNA–membrane hydrophobic interaction can lead to the localization of the bases close to the entrance of the nanopores. Stepwise translocation can also limit the conformational instabilities.

The relatively uncontrollable and arbitrary conformations of the nucleotides affect the ionic current blockade. Nevertheless simulations showed that the dependency of the signal to the nucleotide type is stronger than to its conformation.^27^ Hence even in presence of conformational instabilities, single nucleotide differentiation is possible.

#### 2.4.4 Noise in graphene nanopores

The noise characteristics in 2D and long channel nanopores are different. The difference in the noise levels can be readily observed by comparing the time traces measured in graphene and in SiN_*x*_ nanopores (Fig. 3b). The power spectral density (PSD) of the measured current reveals the frequency dependence of the current fluctuation (noise) and is a widely used parameter to characterize the noise. At high frequencies the noise originates from the capacitive coupling of the *cis* and *trans* cells (Fig. 1b). The dielectric properties of the SiN_*x*_ layer – which is used as the membrane in long channel nanopores and as the support for 2D materials in 2D nanopores – largely govern the capacitance between the cells. As the thicknesses of SiN_*x*_ layers are normally comparable in 2D and long channel nanopores, the high frequency noise levels are relatively similar in both cases. Increasing the thickness of the dielectric layer *e.g.* by making SiN_*x*_/quartz stacking (instead of normally used SiN_*x*_/Si) can efficiently minimize the high frequency noise.^17^

The magnitude of the low frequency noise – known as 1/*f* noise – in nanopores in graphene and other 2D materials is normally much larger (up to 100 times) than in long channel nanopores.^13,14,32^ As a source of a considerable reduction in the signal-to-noise ratio, the high amplitude of 1/*f* noise is an important challenge in 2D nanopores. It was demonstrated that for nanopores in SiN_*x*_ membranes the amplitude of the low frequency noise is inversely dependent on the number of ionic charge carriers ∝ 1/*N*:^33^ a principle known as Hooge's relation. However experimental results do not show such a dependency in monolayer graphene nanopores.^32^ The reasons behind this discrepancy are not yet understood.

The origin of the high-amplitude low-frequency noise in 2D nanopores is under debate. Charge fluctuations in the membrane are potentially capable of inducing noise in the ionic current. Protonation and de-protonation of the carboxyl groups formed at the nanopore rim may cause such fluctuations and can be triggered by varying the pH of the environment. However experimental results did not confirm any strong dependency of the noise to the pH of the buffer solution.^32^ We note that those experiments were performed at relatively high KCl concentration (1 M); more decisive conclusions could be drawn by lowering the ionic strength of the buffer solution.

The incomplete wetting of the hydrophobic graphene has been hypothetized as the source of the measured noise.^4^ To cope with this effect, the hydrophobicity of the graphene membrane was modified by a rapid UV/ozone exposure, yielding graphene oxide. The improved wetting of the membrane on the cost of inducing defects, however, did not lead to a remarkable reduction of the noise level. Importantly, the evaporation of a hydrophilic TiO_2_ layer on graphene considerably reduced the noise. The idea of covering the membrane with hydrophilic materials was followed later by^20^ in which relatively thick membranes of multi-stacked graphene and Al_2_O_3_ layers were made. The top hydrophilic Al_2_O_3_ layer was supposed to provide adequate wetting during the experiments. Indeed very low 1/*f* noise, comparable with long channel nanopores and considerably less than in pure 2D nanopores was measured.

Mechanical instability of the membranes is another parameter which is suspected to cause the noise: the fluctuation of the membrane may lead to the fluctuation of the ion flux. Increasing the thickness of the membrane is an approach to improve its mechanical stability. Indeed prominent trends in lowering the 1/*f* noise level while increasing the thickness (*i.e.* the number of layers) of graphene and h-BN membranes were measured.^17,32^ Such results point out the effect of the mechanical fluctuations as an important source of low frequency noise in 2D nanopore devices. In fact conventional solid state membranes (normally thicker than 20 nm) are mechanically more rigid and stable compared to few layer 2D materials. We note that unlike the interpretation of early reports,^4^ the reduced noise level measured by covering graphene with other materials^4^ or in stacked systems^20^ can be explained in terms of the increased mechanical stability, rather than the modification of the hydrophobicity of the graphene membrane. Minimizing the area of the free-stranding membrane is an approach to improve the stability of the membrane without affecting the surface properties. In fact reduced noise levels have been reported in two samples with very small free standing graphene areas.^6^

Comparing the approaches to improve the mechanical stability of the membrane we note that minimizing the SiN_*x*_ opening blocks a large amount of the pin-holes in graphene which would result in an enhanced signal. The important disadvantage of using thick graphene layers or of depositing other materials is that the increased thickness of the membrane eludes the mono-atomic nature of graphene which potentially provides single base resolution.

### 2.5 Alternative detection approaches

The challenges to improve the resolution of DNA detection have been discussed in the previous section. Fast translocation speed and high noise levels are among the challenges directly linked to the conventional measurement scheme of the nanopores. Hence researchers developed alternative detection methods to avoid these limitations.

**Fig. 5:**
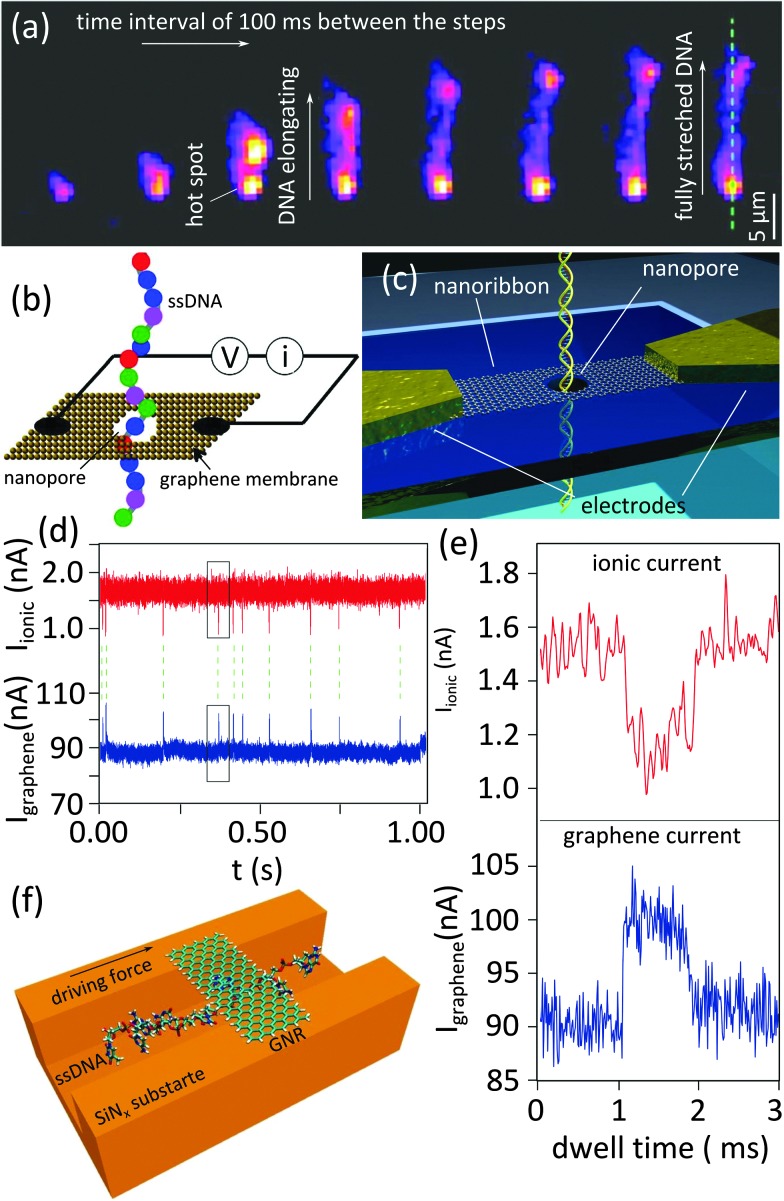
Alternative detection methods based on 2D nanopores. (a) Subsequent snapshots obtained using a confocal scanning fluorescence microscope showing the translocation of a λ-DNA. Adapted with permission from ref. 22. Copyright 2014 American Chemical Society. (b) Proposed electrical scheme for detecting single nucleotides of a ssDNA passing through a graphene nanopore by probing electronic current across the graphene; Adapted with permission from ref. 34. Copyright 2010 American Chemical Society. (c–e) Graphene nanoribbon used for the electrical detection of the translocation of a DNA strand through a nanopore sculpted in the nanoribbon, (c): the schematic representation of the fabricated device, (d): time series of the synchronized measurement of DNA translocations (ionic current through the nanopore and electrical current through the nanoribbon) (e): zoomed-in view of a single translocation event marked inside the boxes in (d). Adapted with permission from ref. 12. Copyright 2013 American Chemical Society. (f) Schematic representation of the proposed nanochannel device promoting DNA–graphene interaction for single nucleotide sequencing; Adapted by permission from Macmillan Publishers Ltd: *Nat. Nanotechnol.*,^36^ copyright 2011.

An optical method was successfully adjusted to measure translocating molecules in a 2D nanopore.^22^ Fluorescently-tagged DNA molecules were electrostatically driven through a nanopore in a graphene membrane made by photothermal sculpting (see the fabrication section, Fig. 2e). The water immersed objective lens of a fluorescent microscope, focused on the nanopore, can be used to monitor and record the dynamics of the DNA translocation. Gold nanoparticles serve as antennas to enhance the fluorescence signal, as they possess a surface plasmon resonance peak matching the absorption peak of the fluorescence dye. Subsequent snapshots in Fig. 5a illustrate the dynamics of the DNA translocation through the nanopore from the moment it enters the nanopore till the moment it is nearly fully stretched (∼18 μm in length). Illustrated by a brighter spot, the fluorescence intensity close to the antenna (marked by the red arrow in the last snapshot) is four to five times higher than at the center (blue arrow), showing the plasmonic resonance effect.

Another novel technique for DNA sequencing was theoretically investigated.^34,35^ The device contains a graphene nanoribbon containing a nanopore supported by a hard material *e.g.* SiO_2_ (Fig. 5b). Simulations showed that passing through the nanopore, each nucleotide would generate currents in the graphene nanoribbon due to the doping effect. Indeed, the electrostatic interaction between the graphene nanoribbon and translocating nucleotides modulates the conductance of the nanoribbon. The conductance spectrum of each individual nucleotide is unique and sufficiently different for each of the four nucleotides. The calculations predict that the detected current is independent of the orientation of the bases, which is an important requirement for the generalization of the approach. The sensitivity of the device inversely depends on the density of the available electronic states in the graphene nanoribbon: the wider the ribbon, the higher the density of states and lower the sensitivity. The efficiency of the scheme for detecting single DNA molecules was probed experimentally by correlating ionic current (typical nanopore application) and electrical current through the nanoribbon (Fig. 5c to e).^12^ Unlike the ionic current in which blocking the nanopore ends up with drops in the ionic current, both drops and peaks can be measured in electrical current due to the ambipolar nature of graphene. Strong correlation between ionic and electric current measurements was achieved.

As a result of the monoatomic channel length of 2D nanopores, the molecule–nanopore rim interaction is very small in 2D nanopore devices which can result in stochastic motion of the molecules through the nanopore. Indeed conformational instability as a result of the stochastic motion overlaps the distribution of the signals from different nucleotides. To cope with this problem, a large modification in the nanopore architecture was proposed.^36^ The proposed device, schematically shown in Fig. 5f composed of a nanoscale fluidic channel locally covered by a graphene nanoribbon bridge. The combination can be regarded as a long nanopore through which DNA molecules – driven by an external field – translocates. The bridge grips the translocating nucleotides firmly by temporarily forming π–π interaction. Hereby, the conformational fluctuation of the nucleotides is limited. The simulations showed that the distinct doping effects of the stacked nucleotides can be measured as electrical signals across the graphene nanoribbon and be used to discriminate between the nucleotides. The possibility of multi-stacking nucleotides to graphene nanoribbon is an important limitation of the technique.

### 2.6 MoS_2_ as an alternative to graphene

Most of the research on 2D nanopores so far has been performed using graphene membranes. Molybdenum disulfide (MoS_2_) is another 2D material with appealing properties in the field. Contrarily to graphene, MoS_2_ has a band gap of 1.8 eV which is demanded for sensing applications. The mobility of MoS_2_ is yet inferior to graphene: this limitation can be overcome by engineering the environment of the device.

The considerably large noise in graphene nanopores has been attributed to the low mechanical instability of the membrane as a result of its atomic thickness. Indeed simulations predict that the noise level reduces by using trilayer graphene membrane of ∼1 nm thickness.^37^ A monolayer MoS_2_ is ∼1 nm thick and is relatively (three times) thicker than a monolayer graphene. Hence on a cost of the slight reduction of the resolution of the device, higher signal to noise ratio is predicted in the 2D nanopores in MoS_2_ membranes. In an early work, a signal to noise ratio of 10 has been measured for 2D nanopores in MoS_2_ which is almost three times higher than what can be achieved in graphene 2D nanopores.^14^

DNA–membrane interaction is another important parameter to select the right material. Molecular dynamics simulations show that in contrary to the graphene, DNA does not stick significantly to the surface of MoS_2_, even without any additional surface treatments.^26^ This result is in agreement with experiments.^14^ Indeed, the presence of the hydrophilic Mo sites in the structure of the MoS_2_ membranes reduces the DNA–membrane interaction. In contrary to MoS_2_, experimentations with h-BN membranes demonstrate remarkably higher interaction with DNA molecules (in comparison to SiN_*x*_ and graphene^6^) even at strong alkaline and salty condition (pH = 10, KCl concentration of 3 M).^13^

The electrically conducting nature of MoS_2_, its low interaction with DNA and the high enough mechanical stability which a free standing MoS_2_ membrane promise a good replacement for graphene in nanopore applications.

### 2.7 Conclusion

Nanopore technology offers a fast, cheap and easy solution for biomolecule sensing. The implementation of 2D materials can potentially improve the resolution of the devices to reach single nucleotide resolution. The possibility of chemically and electrically functionalizing the 2D membranes opens new routes for the customization of the sensors. Only a few methods have been utilized to drill nanopores in 2D materials. Among them, the most robust is to use a beam of energetic electrons combined with *in situ* heating. For large-scale production of sensors, however, significant research efforts are still needed: although TEM is great for proof-of-concept experiments, it is not viable for scalable applications.

DNA sequencing with 2D nanopores met important challenges. The very rapid translocation of the nucleotides through the nanopore is far beyond the sensitivity of the ionic current. Several approaches can be taken to slow down DNA. Separately, the large 1/*f* ionic current noise which severely affects the signal to noise ratio, is another major challenge, very particular to 2D nanopores. The origin of this noise is not entirely clear yet, although the existing reports point the mechanical fluctuations of the membrane as the most probable source. Improving the mechanical stability of the membrane by reducing the area of freestanding part seems to be the most efficient technique to overcome the limitation. Utilizing thicker 2D materials (*e.g.* MoS_2_) is another potential approach.

Alternative detection schemes – not necessarily based on the ionic conduction – have been proposed to overcome the existing difficulties. The conducting nature of 2D materials with externally tunable conductivity is the important supplement of graphene and some other 2D materials to develop such methods. In principle, schemes based on the electronic conduction could be faster than the existing measurement approach based on the ionic current. Theoretical propositions and experimental results of such schemes will be discussed in details in the next section.

## 3 Tunneling current for single molecule detection

**Fig. 6:**
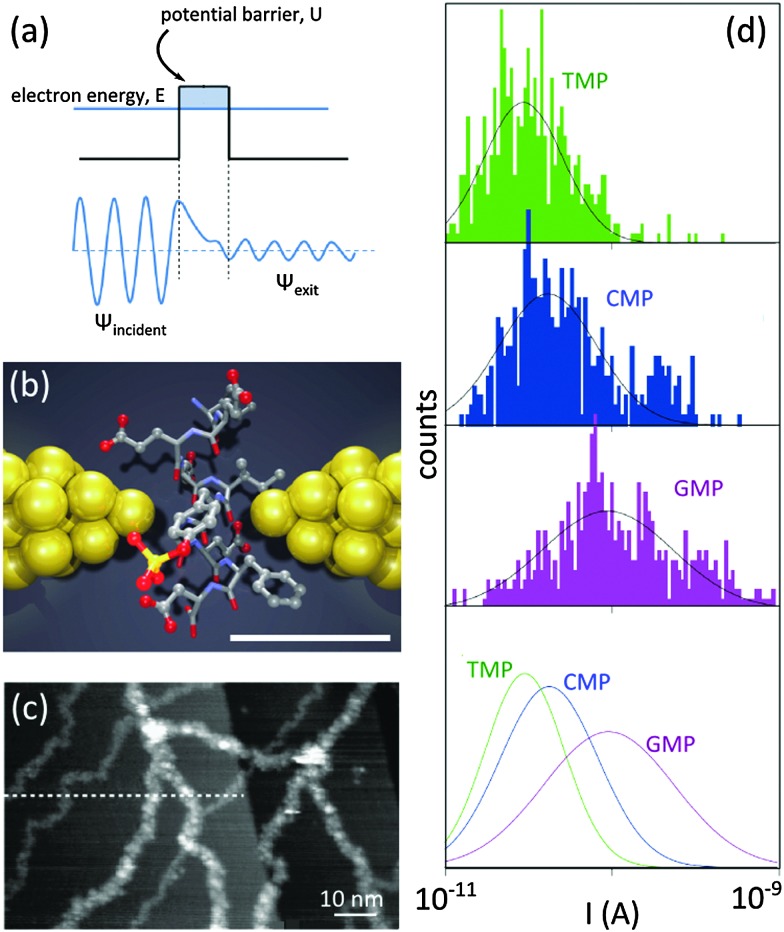
Tunneling current for single-molecule detection. (a) Individual electron tunneling through a rectangular potential barrier: *ψ* is the wave function of the tunneling electron. (b) Schematic illustration of a nanogap between metallic electrodes (*e.g.* break junction) for single-molecule detection: two electrodes (yellow) are separated by a subnanometer-sized gap in which a peptide molecule is placed. Electronic properties of the peptide affect the tunneling current between the electrodes. The scale bar shows 5.5 Å. Adapted by permission from Macmillan Publishers Ltd: *Nat. Nanotechnol.*,^44^ copyright 2014. (c) Tunneling current for the visualization of dsDNA molecules using STM: DNA molecules are visible as white strands. Reprinted from ref. 41, copyright 2003, with permission from Elsevier. (d) Current distributions for thymidine 50-monophosphate (TMP), cytidine 50-monophosphate (CMP) and guanosine 50-monophosphate (GMP) measured by using a break junction with a gap of ∼1 nm. The distribution of the measured current for each nucleotide is unique. Adapted by permission from Macmillan Publishers Ltd: *Nat. Nanotechnol.*,^43^ copyright 2010.

The development of alternative low-noise and fast measurement platforms turned out to be essential to realize biomolecule sequencing with 2D materials. Scanning biomolecules from head-to-tail by measuring the tunneling (transverse) current between two electrodes – separated by a nanogap – as the biomolecule slides through is a remarkable proposition. In classical mechanics, an electron with energy *E* would be unable to overcome the potential barriers with a higher energy *U* (*U* > *E*). In quantum mechanics, however, electrons can tunnel through the barrier with a non-zero probability, yielding a measurable tunneling current^38^ (Fig. 6a). Increasing the barrier length exponentially decreases the probability of electron tunneling.

The detection and sequencing of single molecules could be implemented in a simple device composed of two electrodes separated by a small gap through which a biomolecule would translocate (Fig. 6b). Upon applying an electrical potential, a current would be established between the electrodes, tunneling through the translocating biomolecules. For DNA, the different nucleotides composing the translocating DNA strand – as they possess distinct local density of electronic states and different molecular sizes – were predicted to yield tunneling currents with intensities significantly different for each nucleotide.^39,40^ Separately, the method potentially provides temporal resolution that would allow recording several data points per nucleotide. As the nucleotides pass one-by-one across the gap, recording the tunneling current over time should, in principle, resolve the particular sequence of the nucleotides in the strand.

### 3.1 Experimental detection of single molecules with tunneling current

Tunneling current for the detection of DNA molecules was for the first time exploited in scanning tunneling microscopy (STM). There, a sub-nanometer distance between the tip and the conducting substrate can be realized and maintained using a ‘feed-back loop’. In an early experiment, DNA molecules were imaged individually with STM.^41^ The modulations of the tunneling current between the STM tip and the substrate allowed visualizing the DNA molecules (Fig. 6c).

Molecular break junction is another platform employing the principle of tunneling current for detection. Smoothly pulling the two ends of a metallic rod (*e.g.* a thin wire) yields, in time, a thinner and thinner wire, a few atoms wide junction, a single atom junction, and finally a gap (Fig. 6b). The shape and the width of the gap are controlled by adjusting the applied force on the rod: metallic electrodes with nanogaps as narrow as ∼5 Å have been achieved with this technique.^42^ In a remarkable experiment, a gold break junction was immersed in aqueous solutions containing nucleotides. Three nucleotides, namely thymidine 5′-monophosphate (TMP), guanosine 5′-monophosphate (GMP) and cytidine 5′-monophosphate (CMP), were probed.^43^ In each case, unique tunneling current signatures were detected (Fig. 6d). Similarly, a recent work confirmed the efficiency of the break junction method for the identification of twelve (out of 20) different amino acids, including the detection of post-translational modifications of single peptide molecules.^44^

STM and break junction methods have been studied extensively for various purposes in the past and offer a rather simple and fast solution to investigate biomolecules with tunneling current. However remarkable limitations exist, preventing their direct use for sequencing applications: STM performs best at low temperature (few Kelvins) and ultrahigh vacuum which is not adequate for biological specimens; break junctions do not provide *per se* a fluidic channel to guide long stretches of unfolded biomolecules through the gap. Additionally, the formation of parallel conducting channels reduces the resolution of the techniques in water. Such limitations can be overcome by employing so-called graphene in-plane electrodes.

### 3.2 In-plane graphene nanogap electrodes

The application of graphene for DNA sequencing with tunneling electrons has been theoretically studied. The presence of highly mobile charge carriers is the most distinctive property of graphene over the other 2D materials. Additionally, freestanding graphene membranes are of relatively high mechanical strength and as opposed to metals, each carbon atom is covalently bonded and conjugated to each other. 2D materials can also stand against large transmembrane pressures in liquid environments.^3–5^ As opposed to metallic break junctions, in an ultimate nanogap design, the surface of graphene could be covered with insulating materials, blocking any path for parallel conduction. We note that unlike the 2D nanopore systems, the capacitance between the *cis* and *trans* cells does not influence the tunneling current, hence a lower high-frequency noise is expected.

**Fig. 7:**
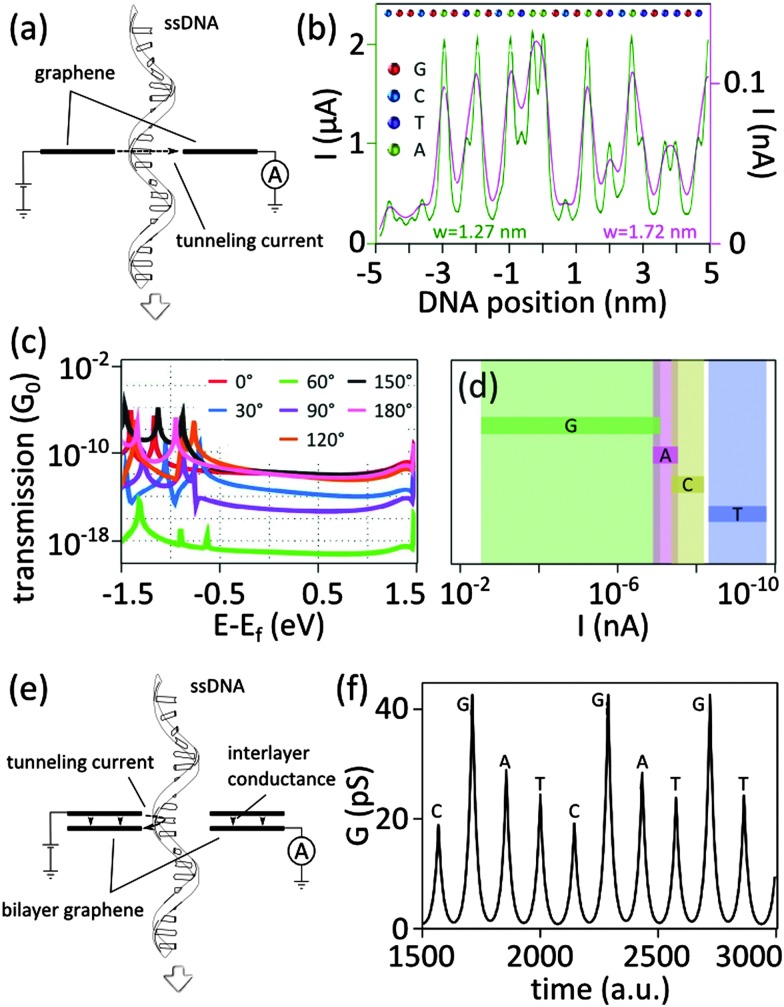
(a) Tunneling current for DNA sequencing with graphene electrodes. (b) Simulations of the tunneling current expected for ssDNA with the sequence CGG CGA GTA GCA TAA GCG AGT CAT GTT GT between two graphene electrodes in a nanogap configuration of two different widths (*w* = 1.27 nm and *w* = 1.72 nm). The different bases are represented with different colors (G in red, C in blue, T in purple, and A in green). Adapted with permission from ref. 39. Copyright 2010 American Chemical Society. (c) Theoretical calculations of the effect of the angle of rotation of deoxyadenosine monophosphate (dAMP) on the zero-bias transmission spectra of dAMP translocating in a graphene nanogap: the different curves correspond to different angles of rotations of dAMP with respect to the graphene edge. Adapted with permission from ref. 40. Copyright 2011 American Chemical Society. (d) Possible ranges of tunneling current for dAMP, dTMP, dGMP, dCMP nucleotides translocating through a nanogap in graphene (with diverse orientation and proximity to the electrodes): calculations were done under an applied bias of 1 V and for a gap width of 1.47 nm. Adapted with permission from ref. 40. Copyright 2011 American Chemical Society. (e) Illustration of the concept of DNA sequencing with tunneling current with a nanopore in bilayer graphene: a DNA molecule translocates through the nanopore while recording the current tunneling from the rim of the nanopore in the top graphene layer to the rim of the nanopore in the bottom graphene layer. (f) Tunneling conductance calculated for ssDNA molecule passing through a nanopore in bilayer graphene (see panel (e) for an illustration of the working device). The amplitude of the rim-to-rim tunneling current signs for individual nucleotides. The figure is adapted from ref. 47.

The concept of using in-plane graphene electrodes for DNA sequencing (Fig. 7a) was investigated theoretically by Henk Postma in 2010:^39^ while a nanogap width of less than 1.5 nm (a distance narrow enough for detecting a transversal tunneling current and large enough for a DNA molecule to be able to translocate) would yield specific tunneling current signatures for each nucleotide, small metric variations in the width of the nanogap drastically impact the measured current. Important parameters that influence the detected signal and are crucial for achieving the single nucleotide resolution will be discussed in details in the next sections.

#### 3.2.1 The width of the nanogap

Considering a set of *N* bases in a DNA strand (random composition of A, T, C and G bases) translocating through a graphene nanogap, the measured current can be expressed as function of the position of the center of the DNA molecule (*x*_0_) with respect to the gap, and the applied bias voltage (*V*):^39^
2
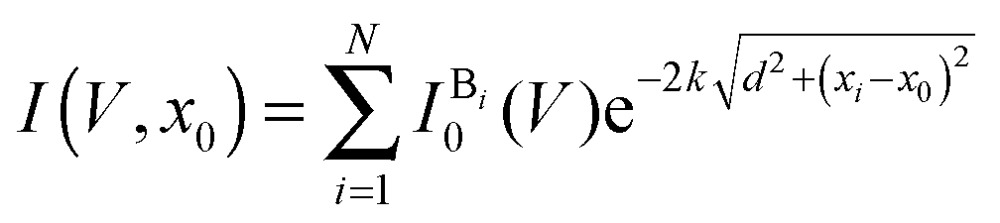
where, *d* is the width of the gap, *x*_*i*_ is the position of the base *i* along the backbone, 
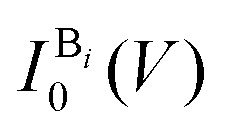
 is a base type-dependent coefficient (B = A, T, C, G) and *k* is the decay constant which depends on the work function of graphene. The results of the simulation for a specific sequence of the bases and for two different gap sizes are shown in Fig. 7b. Clearly, the translocation of each nucleotide through the gap leads to a sharp spike in the calculated current. The amplitude of each spike is different for the different bases and can be considered as the signatures of each nucleotide. Enlarging the tunneling distance by widening the nanogap largely affects the tunneling current: a small increase in the width of the nanogap from 1.27 nm to 1.72 nm yields broader current peaks (in time) and dramatically reduces the intensity of the tunneling current. As a result, the sensitivity of the sensor reduces which adds a lot of difficulties for identifying single bases. The simulations, however, do not take into account the presence of water and ion molecules in the gap.

#### 3.2.2 Conformational uncertainties of DNA in a nanogap

The nucleotides passing through a nanogap may take many different orientations and arbitrary distance to the graphene electrodes.^40^ By means of the density functional theory (DFT), the tunneling currents associated to the four nucleotides, namely deoxyadenosine monophosphate (dAMP), deoxythymidine monophosphate (dTMP), deoxyguanosine monophosphate (dGMP), and deoxycytidine monophosphate (dCMP) in a nanogap between graphene electrodes were simulated.^40^ Size-wise, the nucleotides can be classified in two categories: purine-based (G, A) and pyrimidine-based (C, T) nucleotides. The pyrimidines have a smaller size than purines hence the distance between the nucleotides and the graphene edge is larger for pyrimidine-containing nucleotides. The discrepancy of the sizes affects the transmission (the probability that an electron passes through the potential barrier) spectra. The transmission spectra associated to the dAMP, oriented in several angles with respect to the edge of graphene is plotted in Fig. 7c. The spectra for the other nucleotides were also calculated (not included in this figure). The results showed that for all the studied rotation angles and for different electron energies, the zero bias transmission of purine-based nucleotides ranges from 10^–20^ to 10^–6^ *G*_0_ which is different from pyrimidine-based nucleotides, ranging from 0 to 10^–8^ *G*_0_. The transmission peaks also provide distinctions between the nucleotides: the first peak below the Fermi energy (*E*_f_, the energy of the highest occupied molecular orbital) of the electrodes is found to be in resonance with the HOMO (the highest occupied molecular orbital) of the nucleotide and is therefore dependent on the type of nucleotide. For all the studied orientations, the width of the resonance peak ranges between ∼0.05–0.10 eV for pyrimidines and between ∼0.1–0.2 eV for purines: the size of the nucleotides (at least the graphene–nucleotide coupling) provides means for distinguishing the two categories of nucleotides. The next challenge would therefore be distinguish between the nucleotides within each group (*i.e.* dGMP *vs.* dAMP; and dCMP *vs.* dTMP). The position of the resonance peak is, however, different for dGMP and dAMP. For dGMP, the peak is located close to the Fermi energy of the electrodes (*E* – *E*_f_ ≤ 0.5 eV) while it is far away for the dAMP (*E* – *E*_f_ ≥ 0.5 eV). All the discussion so far was based on the zero biased transmission function. The results are summarized in Table 4. Biasing the electrodes with a finite potential provides tunneling currents with different ranges which allows for further discriminating between the nucleotides, including between dCMP and dTMP (Fig. 7d). The possible current ranges for different nucleotides follow the general order *I*_dGMP_ > *I*_dAMP_ > *I*_dCMP_ > *I*_dTMP_ with very small overlaps.

**Table 4:** Summary of the important features in the calculated zero-bias transmission spectra of different nucleotides^40^

	Purine based nucleotides		Pyrimidine based nucleotides	
	dGMP	dAMP	dTMP	dCMP
Transmission^*a*^	10^–20^–10^–6^ *G*_0_	10^–20^–10^–6^ *G*_0_	0–10^–8^ *G*_0_	0–10^–8^ *G*_0_
Width of the peak	Broad ∼0.1–0.2 eV	Broad ∼0.1–0.2 eV	Narrow ∼0.05–0.10 eV	Narrow ∼0.05–0.10 eV
Position of the peak	Close to Fermi level *E* – *E*_f_ ≤ 0.5 eV	Far from Fermi level *E* – *E*_f_ ≥ 0.5 eV	Far from Fermi level *E* – *E*_f_ ≥ 0.5 eV	Far from Fermi level *E* – *E*_f_ ≥ 0.5 eV

^*a*^Measured for the energy range of –1 eV < *E* < 1 eV.

Functionalization of the electrodes is an effective approach to minimize the conformational uncertainty. The effect of the hydrogen bonds formed in between single nucleotide from a single-stranded DNA and the individual gold atoms at the electrodes (functionalized by purine and pyrimidine molecules) were investigated theoretically.^45^ The results showed a giant improvement in the sensitivity of the device: more than one order of magnitude difference in tunneling current between the nucleotides. Furthermore, the stabilization of the DNA bases against any possible thermal fluctuations dramatically reduced the electrical noise. Slowing down the translocation of DNA is another benefit of generating hydrogen-bonding interactions between the translocating nucleotides and the nanogap electrodes. Applying a large bias voltage can lead to similar results. Indeed, the transverse electric field established between the biased electrodes can be very large and stronger than the driving transmembrane field.^46^ The resultant stabilization of the translocating DNA preserves the sensitivity of the readout for different bases (distinguishability).

#### 3.2.3 Sensitivity of the nanogap

Thermal vibration of the graphene membrane influences the measured signal. The amplitude of the vibrations in few-layer graphene membrane in vacuum may reach 0.16 nm (for a membrane with thickness of 0.6 nm and length of 500 nm)^39^ which is yet smaller than the separation between the bases. In other words, even though the mechanical fluctuations add some noise to the measured signal, high enough sensitivity for sequencing can still be achieved. We note that the thermal vibration of a monolayer graphene in water has not been studied yet.

In the most simple design of a nanogap (Fig. 7a), the surface of the graphene electrodes is in contact with the buffer solution. A parallel ionic current may flow in between the electrodes and reduce the sensitivity of the signal. Covering the graphene with a self-assembled monolayer or with deposited materials can reduce this parallel current. The small contribution of the parallel current between unpassivated carbon atoms at the edges can be minimized by a calibration measurement before and after each DNA translocation.

### 3.3 Bilayer graphene electrodes and interlayer conductance

The fabrication of very narrow graphene electrodes separated by a nanometer gap is very challenging experimentally which has limited the realization of such a device. To by-pass this challenge, a novel device architecture composed of a nanopore in a bilayer graphene was proposed^47^ (Fig. 7e). Both graphene layers are electrically connected. In such a configuration, each nucleotide of a DNA molecule translocating through the nanopore would form a conducting channel between the edges of the graphene layers (*i.e.*, the rim of the nanopore). The current tunneling between the edges of each graphene layer (at the nanopore) would allow identifying the nucleotides. Simulations demonstrated that, indeed, the different nucleotides can be distinguished (Fig. 7f). Yet, the interlayer conductance in between graphene planes (Fig. 7e) is a major limitation.

In a real device the current in between the large layers of graphene can be several orders of magnitude larger than the conductance through the edges of the nanopore. In theory, the interlayer conduction depends on the relative orientation angle in between the layers. Therefore, the two layers can be electrically decoupled at some certain twist angles.^47,48^ The twisted bilayer graphene can be grown chemically, however the twist angle is not controllable. Yet the implementation of a sequencing device based on bilayer graphene would still require a solid (experimental) protocol for the fabrication of well-defined and tunable twisted bilayer graphene. Deposition of the electrodes on one layer without electrically touching the other one is another fabrication challenge.

Using the insulating few- (mono-) layer hexagonal boron nitride (h-BN) in-between graphene layers reduces the interlayer conductance.^49^ It is postulated that in the presence of the molecule in the nanopore, the transmitted current through the two possible paths (i) *via* the molecules, and (ii) *via* the h-BN layer, may show quantum interference which could further improve the sensitivity of the device.^49^ The relative orientation (stacking) of the graphene/h-BN/graphene crystals affects the electrical isolation of the graphene layers. We note that the separation of the two graphene electrodes by the h-BN layer increases the tunneling length, decreasing the tunneling current. Separately, increasing the tunneling channel length also implies that more nucleotides may contribute to the tunneling current (at least if more than one layer of h-BN are used to separate the two graphene electrodes) which decreases the resolution of the measurement.

### 3.4 Conclusion

Several studies (theoretical) showed that tunneling current is sensitive to the molecular composition of translocating biomolecular strands. In comparison to the ionic current, the tunneling current could be potentially more sensitive to alternations in the chemical structure of the nucleotides. Unlike the nanopore scheme, the transmembrane capacitance does not directly affect the tunneling current; hence low amplitude of high-frequency noise is predicted. Indeed, the theoretical work and recent experiments with metallic break junctions show that it is possible to detect subtle differences in chemical compositions using tunneling current (nucleotides and amino acids).

The complex fabrication of the electrodes with a narrow gap (<1.5 nm) is certainly the most important challenge which has prevented the realization of graphene based in-plane electrodes sensors so far. Additionally, theoretical works confirmed the efficiency of the detection scheme for single stranded DNA. As such, ssDNA must first translocate through the nanogap in an unfolded conformation, for example in the presence of unfolding agents such as urea. Separately, a nanopore in a bilayer graphene intrinsically has a physical gap in between the two layers, which could be used also to distinguish nucleotides in a DNA strand.

## 4 General conclusion

Nanopore technologies in solid-state materials – particularly graphene and other 2D materials – are still far from reaching the goal of sequencing long stretches of single-stranded DNA molecules primarily because the conventional measurement scheme based on ionic conduction has not much more advantages over what is currently pursued with biological nanopores. Additionally, the transmembrane capacitance produces a high frequency noise in the same frequency regime as the sequence information. Using low-pass filters to cut-off the noise also cuts-off the sequencing information, unfortunately. The situation is even worse in 2D graphene nanopores since a high level of low frequency 1/*f* noise always circumvent the data acquisition: the low mechanical stability of 2D membranes seems to be the origin of this noise. And importantly, to reach potential applications of 2D nanopores for DNA or other biomolecular sequencing applications, the poor scalability of the techniques developed so far to fabricate 2D nanopores have to be thought through.

Nanogap in 2D materials is a proposition which potentially can overcome some of the sequencing challenges. Their fabrication is, however, even more complicated than nanopores. While the tunneling current has been predicted to be very sensitive to the available electronic states in nucleotides and therefore may provide more accurate readings, all predictions are based on theoretical work (except for some of peculiar experimental studies with break junction). Certainly, the transmembrane potential used to drive the molecules could be isolated further from the measurement circuit to limit the high frequency noise. The electrodes could also be supported on relatively thick membranes providing higher mechanical stability.

Very importantly, small nanogaps with a width of less than ∼1.5 nm are also required to establish a measureable tunneling current. While high temperature STEM techniques showed the feasibility for the fabrication, tunneling has not been measured so far with graphene for example. Additionally, tunneling currents are very sensitive to the gap width and only a few percent increase in the gap width dramatically reduce the signal by orders of magnitude. Additionally, conventional nanofabrication methods are hardly capable to produce such short nanogaps, explaining why the idea has not been realized yet experimentally.

An alternative to longitudinal nanogaps was proposed theoretically: the distance between the layers in a bilayer graphene can be regarded as a natural gap between the two basal plane graphene electrodes. This proposition may ease the fabrication in the sense that the gap can be achieved naturally and reproducibly. However the isolation of the two conducting layers is hard to achieve, even in twisted graphene bilayers.

To conclude, the approaches to sequence DNA with 2D materials are still at a very early stage. The existing nanofabrication methods failed in realizing sequencing. But the principle is still viable: an atomic layer of a 2D material – particularly a graphene edge – can scan a genome and record its sequence as DNA is scanned. It is now clear that developing unconventional fabrication methods are vital (particularly the one that does not need a transmission electron microscope, nor a cleanroom).

It is hard to predict now whether 2D crystals will ever meet the goal of DNA sequencing one day. Certainly, however, in the quest of this goal, new science will be discovered setting out even more horizons for the research.
